# Household environmental tobacco smoke and risks of asthma, wheeze and bronchitic symptoms among children in Taiwan

**DOI:** 10.1186/1465-9921-11-11

**Published:** 2010-01-29

**Authors:** Ching-Hui Tsai, Jiun-Hau Huang, Bing-Fang Hwang, Yungling L Lee

**Affiliations:** 1 Institute of Preventive Medicine, College of Public Health, National Taiwan University, Taipei, Taiwan; 2 Institute of Health Policy and Management, College of Public Health, National Taiwan University, Taipei, Taiwan; 3 Department of Occupational Safety and Health, College of Public Health, China Medical University, Taichung, Taiwan; 4 Research Center for Genes, Environment and Human Health, College of Public Health, National Taiwan University, Taipei, Taiwan

## Abstract

**Background:**

Although studies show that maternal smoking during pregnancy increases the risks of respiratory outcomes in childhood, evidence concerning the effects of household environmental tobacco smoke (ETS) exposure remains inconsistent.

**Methods:**

We conducted a population-based study comprised of 5,019 seventh and eighth-grade children in 14 Taiwanese communities. Questionnaire responses by parents were used to ascertain children's exposure and disease status. Logistic regression models were fitted to estimate the effects of ETS exposures on the prevalence of asthma, wheeze, and bronchitic symptoms.

**Results:**

The lifetime prevalence of wheeze was 11.6% and physician-diagnosed asthma was 7.5% in our population. After adjustment for potential confounders, *in utero *exposure showed the strongest effect on all respiratory outcomes. Current household ETS exposure was significantly associated with increased prevalence of active asthma, ever wheeze, wheeze with nighttime awakening, and bronchitis. Maternal smoking was associated with the increased prevalence of a wide range of wheeze subcategories, serious asthma, and chronic cough, but paternal smoking had no significant effects. Although maternal smoking alone and paternal smoking alone were not independently associated with respiratory outcomes, joint exposure appeared to increase the effects. Furthermore, joint exposure to parental smoking showed a significant effect on early-onset asthma (OR, 2.01; 95% CI, 1.00-4.02), but did not show a significant effect on late-onset asthma (OR, 1.17; 95% CI, 0.36-3.87).

**Conclusion:**

We concluded that prenatal and household ETS exposure had significant adverse effects on respiratory health in Taiwanese children.

## Introduction

The reported prevalence of childhood asthma/wheeze is increasing around the world [1-4]. The changing pattern of these diseases has not been fully explained, in part because of an incomplete understanding of its pathogenesis. The change has been too rapid to be accounted for by changes in gene frequencies. It is also unlikely that it can be totally accounted for by changes in either clinical diagnostic patterns or increased recognition of respiratory symptoms by the general population [5]. This shift does, however, suggest a role for environmental exposures in the etiology of this evolving epidemic [6].

It is estimated that around 1.3 billion people worldwide smoke [7], and the number is predicted to increase in the coming years as smoking rates continue to increase among youth, primarily among young girls [8]. Exposure to environmental tobacco smoke (ETS) is common in children and causes substantial morbidity [9-13]. Estimates of population attributable risk for household ETS exposure in children range from 9% for asthma prevalence to 25% for hospital admissions due to respiratory symptoms [13]. The World Health Organization estimates that approximately half of the children in the world are exposed to ETS, mostly in their homes [14]. In Taiwan, schoolchildren are not typically exposed in public due to the legislative ban on public smoking and regular health promotion campaigns; home exposure is likely the dominant source of ETS. It was reported that approximately 60% Taiwanese children under the age of 17 were exposed to household ETS [15], and therefore an estimated 3.3 million children were at risk for adverse health effects from this exposure.

Evidence of the effects of ETS exposure on childhood respiratory outcomes is inconsistent [16-22]. Although an effect for paternal smoking has been reported, exposure to maternal smoking has consistently had the strongest association with adverse respiratory health effects [9,23]. The strength of the association between maternal smoking and asthma/wheeze is in part due to larger ETS doses from maternal smoking than from other sources [24]. Although there is evidence that maternal smoking during pregnancy increases the risk of asthma/wheeze in childhood [11,25-27], the effects of *in utero *exposure on the occurrence of respiratory symptoms have not been extensively studied or compared with other household sources of ETS.

The Taiwan Children Health Study (TCHS) offers an opportunity to investigate the effects of household ETS exposure on the occurrence of respiratory symptoms in Taiwanese children. At cohort entry, we used lifetime tobacco smoke exposure histories to investigate the relationships of multiple sources of ETS exposure with parental-reported respiratory outcomes. The individual and joint effects of parental smoking habits were also examined for associations with early-onset and late-onset childhood asthma.

## Methods

### Study design

The Taiwan Children Health Study (TCHS) has a multi-purpose nationwide design, and is focused on common environmental factors such as outdoor pollutants and household ETS exposure. Communities in Taiwan were selected with the aim of maximizing the variability and minimizing the correlations of exposures to outdoor pollutants based on historic routine air monitoring data. In communities with pollution patterns of interest, neighborhoods with stable, largely middle-income populations were identified from 2004 census data. To address community-level sources of variability, we randomly sought participating communities within existing financial constraints. School district representatives in participating communities were consulted to identify suitable schools, based on demographic stability, likely parental cooperation, and absence of local pollution sources. Our study population finally comprised middle-school children from 14 diverse communities in Taiwan.

To permit cross-sectional assessment of environmental factors, we recruited 350-450 participants from each of the study communities. In each classroom targeted for participation, every student was invited to volunteer. Classroom-level incentives were used to encourage participation. In each school, science, health, or physical education classes were targeted, excluding any special classes for gifted or learning-disabled subjects. The study protocol was approved by the Institutional Review Board at our university hospital, and it complied with the principles outlined in the Helsinki Declaration [28].

### Questionnaire of respiratory health

A total of 5,804 seventh and eighth-grade children were recruited from public schools in 14 Taiwanese communities in 2007. The questionnaire was distributed in all communities simultaneously; subjects were given the forms by project staff following their pulmonary function tests and asked to complete and return them the following day. Questionnaire responses by parents or guardians were used to categorize children's asthma status, age at asthma diagnosis, wheeze, and history of bronchitic symptoms. Children were considered to have asthma if there was a positive answer to the question "Has a doctor ever diagnosed this child as having asthma?" Active asthma was defined as physician-diagnosed asthma with any asthma-related symptoms or illness in the previous 12 months. Serious asthma was defined as ever visit emergency rooms or ever hospitalized. Early-onset asthma was defined as age of onset for asthma before 5 years of age. Late-onset asthma was onset after 5 years of age. Wheeze was defined as any occurrence of the child's chest sounding wheezy or whistling. Current wheeze was defined as wheezing for 3 or more days out of the week for a month or longer in the previous year. Bronchitis status was positive if subjects had a physician-diagnosed episode in the prior 12 months. Chronic cough was defined as cough in the morning or at other times of the day that lasted for three months in a row or more during the prior 12 months. Chronic phlegm was defined by a "yes" answer to the question "Other than with colds, does this child usually seem congested in the chest or bring up phlegm?"

### Environmental tobacco smoke and other exposure

We collected information about the current and past household smoking status of each participant's adult household members and regular household visitors. *In utero *exposure to ETS was defined as maternal smoking during pregnancy. Current number of household smokers, current number of cigarettes smoked inside the house per day, and years of household ETS exposure divided by age were recorded. Mutually exclusive categories of current household ETS exposure were defined as none, paternal smoke only, maternal smoke only, or both paternal and maternal smoke exposure. Personal smoking was defined as a history of smoking more than 100 cigarettes during subject's lifetime, as ascertained by a private interview during pulmonary function tests.

In the baseline questionnaire, we also obtained information on children's sex, age, grade, parental education, number of siblings, gestational age, neonatal special care, personal/family history of atopy, and many residential risk factors, such as pet ownership, incense burning, air cleaner, air conditioner, dehumidifier, and carpet use at home. Occurrences of any severe chest illness, including pneumonia, croup, and other illnesses, before age 2 or after age 2 were dichotomized. Personal history of atopy included any history of hay fever, allergies to food or medicine, inhaled dusts, pollen, molds, animal fur or dander, or skin allergies. Parental history of atopy was defined as any biological parent in whom hay fever or allergies had been diagnosed. Parental history of asthma was defined as any biological parent in whom asthma had been diagnosed.

### Statistical analysis

Unconditional logistic regression models were used to assess the individual and joint effects of ETS exposures on the occurrence of respiratory symptoms. On the basis of study design and a priori consideration of potential confounders, we included age, sex, parental education, parental history of asthma, parental history of atopy, and community in all models. If estimates of ETS effects changed by at least 10% when a covariate was included in the base models, then the covariate was included in the final models. The odds ratios (ORs) for the association of ETS exposures with early-onset and late-onset childhood asthma were computed using a likelihood method for polytomous logistic regression models. Subjects with missing covariate information were included in the model using missing indicators [29]. All analyses were conducted using SAS software version 9.1 (SAS Institute, Cary, NC, USA). Statistical significance was set at p < 0.05 based on two-sided estimation.

## Results

The overall response rate in TCHS was 86.5% (2,432 boys and 2,587 girls and their parents). The majority of participants were 12 years of age and from households with low parental educational levels (Table 1). All subjects were of Han Chinese ethnic origin. 3.1% of children had a parental history of asthma and 25.0% had a parental history of atopy at the time of interview. 9.3% of participants had no siblings. Premature birth occurred for 9.3% of children and 8.6% required neonatal special care (Table S1 in Additional file 1).

**Table 1:** Demographic characteristics and environmental tobacco smoke (ETS) exposure of the study participants

	Total		Boys		Girls	
	
	(n = 5019)		(n = 2432)		(n = 2587)	
	
	n	%	n	%	n	%
Age, yr						
12	3467	69.1	1696	69.7	1771	68.5
13	1234	24.6	577	23.7	657	25.4
14	318	6.3	159	6.5	159	6.1
Parental education, yr†						
≦ 12	3163	63.5	1509	62.7	1654	64.3
13~15	949	19.1	460	19.1	489	19.0
≧ 16	867	17.4	437	18.2	430	16.7
Gestational age†						
Full term	4435	90.7	2113	89.6	2322	91.6
<4 wk early	315	6.4	173	7.3	142	5.6
≧ 4 wk early	142	2.9	71	3.0	71	2.8
Family history of asthma†						
Yes	140	3.1	68	3.1	72	3.1
Family history of atopy*†						
Yes	1257	25.0	641	25.0	643	25.0
Family income**†						
≦ 400,000	1750	37.7	792	35.4	958	39.8
410,000~800,000	1833	39.5	911	40.7	922	38.3
≧ 810,000	1060	22.8	534	23.9	526	21.9
Active smoking						
Yes	37	0.7	28	1.2	9	0.3
*In utero *exposure†						
Yes	197	3.9	82	3.4	115	4.5
Lifetime ETS†						
Yes	2445	49.0	1176	48.7	1269	49.4
Currently ETS†						
Yes	2241	44.9	1078	44.6	1163	45.3
Previous ETS only†						
Yes	204	4.1	98	4.1	106	4.1
Currently amount of ETS***†						
0	2778	55.8	1354	56.2	1424	55.4
≦ 10	1728	34.7	838	34.8	890	34.6
> 10	472	9.5	217	9.0	255	9.9
Percent of ETS****†						
0	2778	55.8	1354	56.3	1424	55.4
≦ 20%	1406	28.3	693	28.8	713	27.7
> 20%	791	15.9	358	14.9	433	16.8
Dad smoking†						
Yes	1794	36.0	835	34.6	959	37.2
Mom smoking†						
Yes	171	3.4	69	2.9	102	4.0
Number of smokers†						
0	2723	54.8	1368	56.8	1355	53.0
1	1476	29.7	695	28.9	781	30.5
2	513	10.3	238	9.9	275	10.8
≧ 3	253	5.1	106	4.4	147	5.7

*Atopy is defined as allergic rhinitis or atopic eczema.

**New Taiwan dollars per year ($1 US = $ 33 New Taiwan).

***Average cigarettes per day

****Average percent of ETS in lifetime

† Number of subjects does not add up to total N because of missing data.

We excluded 37 subjects (0.7%) with active smoking exposure in risk factor determination, due to sample size limitation for stratification analyses. *In utero *exposure to maternal smoking occurred in 3.9% of children, 49.0% had exposure to household ETS at any time during their lives, and 44.9% had current household ETS exposure. The prevalence of paternal smoking was 36.0% and maternal smoking was 3.4% (Table 1). More than 15% of children had two or more smokers at home. *In utero *exposure to maternal smoking and household ETS exposure were highest among children with lower parental education level and among children from low-income families (Table S2 in Additional file 1).

The lifetime prevalence of wheeze was 11.6% and physician-diagnosed asthma was reported in 7.5% of children. In subjects with asthma, about two-thirds were diagnosed before 5 years of age, about one third of cases continued to require medication, and 59 cases (1.3%) had experienced emergency room visits or hospitalization due to asthma attacks within the previous year (Table 2). The prevalence of respiratory symptoms was higher in children with *in utero *exposure or household ETS exposure than in unexposed children (Table S3 in Additional file 1).

**Table 2:** Prevalence of asthma, wheeze and bronchitic symptoms of the study participants

	Total		Boys		Girls	
	
	(n = 4982)		(n = 2404)		(n = 2578)	
	
	n	%	n	%	n	%
Asthma						
Ever asthma	369	7.5	195	8.2	174	6.8
Active asthma	167	3.4	89	3.7	78	3.0
Early-onset asthma*	237	4.9	123	5.3	114	4.6
Late-onset asthma**	120	2.6	64	2.8	56	2.3
Treatments for asthma						
Medication use	122	2.5	65	2.7	57	2.2
ER visit or hospitalization	59	1.3	25	1.1	34	1.4
Wheeze						
Ever wheeze	577	11.6	307	12.8	270	10.5
Current wheeze	181	3.7	88	3.7	93	3.7
Awakened at night	107	2.2	40	1.7	67	2.6
Bronchitic symptoms						
Brochitis	286	5.8	160	6.7	126	4.9
Chronic cough	166	3.3	89	3.7	77	3.0
Phlegm without cold	214	4.3	111	4.7	103	4.0

Number of subjects does not add up to total N because of missing data.

*Early-onset: asthma diagnosed ≦ 5 yr of age.

**Late-onset: asthma diagnosed > 5 yr of age.

To further investigate the different patterns of household ETS effects and *in utero *exposure to maternal smoking on children's respiratory health, we examined the relationships of these variables with subcategories of asthma, wheeze, and bronchitic symptoms (Table 3 and Table 4). After adjustment for potential confounders, we found that *in utero *exposure to maternal smoking was positively associated with all respiratory outcomes, with greater effects on serious asthma, such as emergency room visits or hospitalization within the previous year (OR, 4.33; 95% CI, 2.03-9.24). Current household ETS exposure was significantly associated with increased prevalence of active asthma, ever wheeze, wheeze with nighttime awakening, and bronchitis. Maternal smoking was associated with increased prevalence of a wider range of wheeze subcategories, serious asthma, and chronic cough. Paternal smoking and past-only ETS exposure in the household were not associated with any respiratory outcome, but the presence of three or more household smokers was positively associated with all the subcategories of asthma, wheeze, and bronchitic symptoms. The number of current smokers at home showed significant trends in relationship to serious asthma, ever wheeze, wheeze with nighttime awakening, and chronic phlegm without cold (Table 3 and Table 4). In our cohort, the number of current household cigarettes smoked and the percent of ETS exposure during lifetime also showed increasing trends for risks of respiratory outcomes. When the patterns of ETS effects were stratified by the sex of the child, we found almost all respiratory outcomes showed different, but not statistically significant, effects between boys and girls (Table S4 in Additional file 1). In addition, we found little evidence that the magnitude of the effects of household ETS exposure on respiratory outcomes varied by age, parental education, family income, or number of siblings (data not shown).

**Table 3:** Effects of environmental tobacco smoke exposure (ETS) on subcategories of asthma

		Asthma				Treatments for asthma			
		
		Ever asthma		Active asthma		Medication use		ER visit or hospitalization	
				
		OR	95%CI	OR	95%CI	OR	95%CI	OR	95%CI
ETS									
*In utero *exposure		1.53	(0.95,2.48)	2.06	(1.14,3.70)	1.95	(0.99,3.83)	4.33	(2.03,9.24)
Currently		1.15	(0.92,1.44)	1.39	(1.00,1.93)	1.33	(0.91,1.95)	1.71	(0.98,2.96)
Previous only		0.79	(0.43,1.44)	0.69	(0.28,1.72)	0.56	(0.17,1.79)	NA	
ETS sources									
Dad		1.07	(0.85,1.35)	1.11	(0.79,1.55)	0.99	(0.67,1.47)	1.28	(0.74,2.20)
Mom		1.40	(0.82,2.39)	1.67	(0.84,3.31)	0.99	(0.39,2.52)	3.16	(1.29,7.77)
Number of smokers									
0		1		1		1		1	
1		0.91	(0.71,1.19)	1.05	(0.72,1.52)	1.01	(0.66,1.57)	1.04	(0.54,2.01)
2		0.80	(0.53,1.21)	0.92	(0.51,1.67)	0.76	(0.37,1.56)	0.58	(0.17,1.95)
≧ 3		1.61	(1.04,2.50)	2.28	(1.30,4.01)	2.56	(1.40,4.69)	4.56	(2.20,9.46)
p value for trend		0.47		0.05		0.07		0.004	
Currently amount of ETS*									
0		1		1		1		1	
≦ 10		1.06	(0.83,1.36)	1.26	(0.88,1.80)	1.14	(0.75,1.74)	1.21	(0.65,2.26)
> 10		1.42	(0.99,2.03)	2.02	(1.27,3.24)	2.21	(1.32,3.71)	2.81	(1.38,5.73)
p value for trend		0.10		0.005		0.009		0.01	
Percent of ETS**									
0		1		1		1		1	
≦ 20%		0.99	(0.76,1.29)	1.13	(0.77,1.67)	1.19	(0.77,1.85)	1.12	(0.57,2.18)
> 20%		1.43	(1.07,1.92)	1.79	(1.19,2.69)	1.52	(0.93,2.46)	2.50	(1.32,4.74)
p value for trend		0.04		0.01		0.09		0.01	

Models are adjusted for age, sex, parental education, family history of asthma, family history of atopy, gestational age, and community.

*Average cigarettes per day

**Average percent of ETS in lifetime

**Table 4:** Effects of environmental tobacco smoke exposure (ETS) on subcategories of wheeze and bronchitic symptoms

		Wheeze						Bronchitic symptoms					
		
		Ever wheeze		Current wheeze		Awakened at night		Brochitis		Chronic cough		Phlegm without cold	
						
		OR	95%CI	OR	95%CI	OR	95%CI	OR	95%CI	OR	95%CI	OR	95%CI
ETS													
*In utero *exposure		1.98	(1.35,2.89)	3.21	(1.95,5.29)	3.18	(1.70,5.96)	1.88	(1.11,3.17)	1.99	(1.10,3.60)	2.04	(1.21,3.46)
Currently		1.28	(1.07,1.54)	1.30	(0.96,1.78)	1.64	(1.09,2.46)	1.39	(1.08,1.79)	1.13	(0.82,1.57)	0.97	(0.73,1.30)
Previous only		1.08	(0.69,1.69)	1.36	(0.70,2.65)	0.21	(0.03,1.51)	1.43	(0.82,2.49)	1.56	(0.80,3.04)	1.57	(0.87,2.82)
ETS sources													
Dad		1.09	(0.90,1.32)	0.95	(0.69,1.32)	1.39	(0.93,2.08)	1.01	(0.77,1.31)	0.95	(0.68,1.33)	1.21	(0.91,1.62)
Mom		1.76	(1.15,2.68)	2.7	(1.54,4.75)	2.17	(1.02,4.64)	1.68	(0.94,3.03)	2.39	(1.30,4.39)	1.77	(0.97,3.22)
Number of smokers													
0		1		1		1		1		1		1	
1		0.98	(0.79,1.22)	0.96	(0.66,1.38)	1.39	(0.87,2.22)	0.96	(0.71,1.28)	0.78	(0.53,1.16)	1.02	(0.73,1.42)
2		1.45	(1.09,1.94)	1.53	(0.96,2.42)	1.83	(0.99,3.37)	1.33	(0.89,1.98)	1.00	(0.58,1.72)	1.13	(0.71,1.81)
≧ 3		1.63	(1.12,2.37)	1.43	(0.77,2.66)	3.51	(1.87,6.61)	1.6	(0.95,2.69)	1.93	(1.09,3.42)	2.48	(1.52,4.03)
p value for trend		0.003		0.10		<0.001		0.07		0.20		0.005	
Currently amount of ETS*													
0		1		1		1		1		1		1	
≦ 10		1.20	(0.98,1.46)	1.30	(0.93,1.82)	1.40	(0.90,2.17)	1.33	(1.01,1.74)	0.89	(0.61,1.28)	0.90	(0.66,1.23)
> 10		1.64	(1.23,2.19)	1.43	(0.88,2.34)	2.38	(1.36,4.18)	1.50	(0.99,2.27)	2.20	(1.42,3.42)	1.29	(0.83,1.99)
p value for trend		0.001		0.07		0.003		0.02		0.01		0.58	
Percent of ETS**													
0		1		1		1		1		1		1	
≦ 20%		1.15	(0.93,1.42)	0.95	(0.64,1.40)	1.14	(0.70,1.87)	1.24	(0.92,1.66)	0.64	(0.42,0.99)	0.79	(0.56,1.12)
> 20%		1.56	(1.22,1.98)	2.06	(1.43,2.99)	2.40	(1.49,3.85)	1.66	(1.19,2.31)	2.13	(1.47,3.08)	1.29	(0.89,1.85)
p value for trend		0.001		0.001		0.001		0.003		0.001		0.41	

Models are adjusted for age, sex, parental education, family history of asthma, family history of atopy, gestational age, and community.

*Average cigarettes per day

**Average percent of ETS in lifetime

The risks of respiratory outcomes for maternal smoking alone were generally higher than for paternal smoking alone (Table 5). The effect of maternal smoking and paternal smoking exposure did not vary substantially between boys and girls. Although maternal smoking alone and paternal smoking alone were not independently associated with respiratory outcomes, joint exposure appeared to increase the individual effects of parental ETS on serious asthma (OR, 4.30; 95% CI, 1.57-11.80), ever wheeze (OR, 1.81; 95% CI, 1.09-3.00), current wheeze (OR, 2.74; 95% CI, 1.42-5.29), bronchitis (OR, 1.97; 95% CI, 1.03-3.77), and chronic phlegm without cold (OR, 2.65; 95% CI, 1.39-5.03).

**Table 5:** Joint effects of parental smoking on subcategories of asthma, wheeze and bronchitic symptoms

		Parental smoking						
	
		None	Dad only		Mom only		Both	
				
			OR	95%CI	OR	95%CI	OR	95%CI
Asthma								
Ever asthma		1	1.03	(0.81,1.32)	1.05	(0.36,3.03)	1.60	(0.86,2.96)
Active asthma		1	1.08	(0.76,1.52)	1.62	(0.47,5.50)	1.76	(0.77,4.01)
Treatments for asthma								
Medication use		1	1.01	(0.68,1.51)	1.33	(0.30,5.80)	0.85	(0.25,2.82)
ER visit or hospitalization		1	1.09	(0.60,1.96)	1.45	(0.18,11.50)	4.30	(1.57,11.80)
Wheeze								
Ever wheeze		1	1.06	(0.87,1.29)	1.77	(0.83,3.78)	1.81	(1.09,3.00)
Current wheeze		1	0.85	(0.60,1.20)	2.11	(0.72,6.18)	2.74	(1.42,5.29)
Awakened at night		1	1.39	(0.91,2.11)	3.10	(0.90,10.70)	2.23	(0.85,5.82)
Bronchitic symptoms								
Brochitis		1	0.94	(0.72,1.24)	0.83	(0.20,3.55)	1.97	(1.03,3.77)
Chronic cough		1	0.97	(0.69,1.38)	4.38	(1.84,10.40)	1.55	(0.65,3.68)
Phlegm without cold		1	1.10	(0.81,1.49)	0.37	(0.05,2.77)	2.65	(1.39,5.03)

*Models are adjusted for age, sex, parental education, family history of asthma, family history of atopy, gestational age, and community.

After adjustment for potential confounders, *in utero *exposure to maternal smoking had an OR of 1.67 (95% CI, 0.93-2.99) with asthma diagnosed before 5 years of age and an OR of 1.49 (95% CI 0.69-3.19) with asthma diagnosed after 5 years of age (Table 6). In our population, paternal or maternal smoking alone did not show effects, but joint exposure to parental smoking showed significant effect on early-onset asthma (OR, 2.01; 95% CI, 1.00-4.02). However, joint exposure to parental smoking did not show a significant effect on late-onset asthma (OR, 1.17; 95% CI, 0.36-3.87). Although estimates were imprecise, the effects of current exposure to maternal smoking appeared to be larger in the 'younger age at diagnosis' group. Children with three or more household smokers had a significant risk for early-onset asthma (OR, 2.80; 95% CI, 1.27-6.17).

**Table 6:** Effects of household environmental tobacco smoke (ETS) exposure on asthma, stratified by age at asthma diagnosis

	Early-onset asthma†		Late-onset asthma‡	
	OR	95%CI	OR	95%CI
ETS				
*In utero *exposure	1.67	(0.93,2.99)	1.49	(0.69,3.19)
Currently	1.15	(0.87,1.52)	1.07	(0.74,1.57)
Previous only	0.70	(0.32,1.53)	0.85	(0.31,2.35)
ETS sources				
Dad	1.05	(0.79,1.41)	1.13	(0.77,1.66)
Mom	1.60	(0.85,2.99)	0.92	(0.33,2.58)
Number of smokers				
0	1		1	
1	0.72	(0.42,1.24)	0.74	(0.38,1.46)
2	0.49	(0.22,1.10)	0.86	(0.34,2.17)
≧ 3	2.80	(1.27,6.17)	2.07	(0.73,5.88)
p value for trend	0.75		0.31	
Currently amount of ETS*				
0	1		1	
≦ 10	0.86	(0.56,1.32)	0.82	(0.46,1.44)
> 10	1.51	(0.85,2.66)	1.53	(0.73,3.21)
p value for trend	0.20		0.39	
Percent of ETS**				
0	1		1	
≦ 20%	0.77	(0.50,1.18)	0.86	(0.49,1.52)
> 20%	1.61	(1.02,2.56)	1.34	(0.71,2.51)
p value for trend	0.11		0.46	
parental smoking				
none	1		1	
dad only	1.00	(0.74,1.36)	1.16	(0.78,1.72)
mom only	0.88	(0.21,3.78)	0.71	(0.09,5.34)
both	2.01	(1.00,4.02)	1.17	(0.36,3.87)

Models are adjusted for age, sex, parental education, parental history of asthma, parental history of atopy, gestational age, and community.

† Early-onset: asthma diagnosed ≦ 5 yr of age.

‡ Late-onset: asthma diagnosed > 5 yr of age.

*Average cigarettes per day

** Percent of ETS exposure in lifetime

## Discussion

Our population-based epidemiologic study showed the harmful effects of fetal and current exposure to tobacco smoke products. Prenatal exposure due to maternal smoking had the strongest effects on respiratory symptoms. Current household ETS exposure also showed significant adverse effects, but past-only ETS exposure was not associated with any respiratory outcome. In addition, the number of current household cigarettes smoked, the percentage of ETS exposure during lifetime, and the number of current smokers at home showed increasing trends in risks of respiratory symptoms.

Age, sex, active smoking habits, parental atopic history, and parental education might contribute to asthma and wheeze in childhood [3,30]. We minimized interference from these confounders by recruiting lifelong non-smokers of similar age at study entry, and adjusting potential confounders by regression models. Although maternal smoking was, as expected, a strong determinant of preterm delivery and low birth weight, and these adverse pregnancy outcomes were strong predictors of respiratory problems, only gestational age showed an effect in our study. Adjustment for indoor residential factors resulted in only small changes in the effect estimates, and these covariates were not included in the final models.

In our population, 197 (3.9%) children were reported to have had *in utero *ETS exposure (Table 1). The prevalence is much lower than other Western studies [11,19,25,27,31-34]. *In utero *exposure to maternal smoking showed significant adverse effects on respiratory health, with an adjusted OR of 3.21 (95% CI 1.95-5.29) for current wheeze. In contrast, current exposure to ETS showed a smaller effect, with an adjusted OR of 1.30 (95% CI 0.96-1.78) for current wheeze (Table 3 and Table 4). Our findings on the stronger effect of prenatal exposure compared with current ETS exposure are consistent with the results in the 24 Cities Study [25]. Other studies of Californian[11] and Russian[32] children also provided evidence of the relative importance of prenatal exposure on respiratory outcomes.

These associations of *in utero *exposure with respiratory outcomes are consistent with the evidence that *in utero *exposure adversely affects postnatal pulmonary function and increases the occurrence of respiratory symptoms [19,27,31-34]. Furthermore, *in utero *exposure may also affect the development and maturation of the pulmonary immune system [35]. Inappropriate persistence of a Th_2_-dominant response appears to increase allergic sensitization upon sufficient exposure to a variety of common antigens that underlie the pathogenesis of asthma [36]. Our result is in agreement with the biological plausibility that *in utero *exposure to maternal smoking, through mechanisms of decreasing pulmonary function and increasing bronchial hyper-responsiveness (BHR), induces asthma occurrence, especially during the first five years of life (Table 6).

Exposure at home by parental smoking is likely the most common source of ETS exposure in children. Exposure to household ETS among children has been reported to vary from 27.6% to 77.8% [8]. Our prevalence of 49.0% for lifetime ETS exposure and 44.9% for current ETS exposure are similar to many Western countries, but are far lower than the prevalence of 80.0% reported in a recent study in a Chinese population [22]. While a review of epidemiologic studies on allergies has been inconclusive [16-22], murine model and human experimental studies may explain the findings of the present investigation. In our results, current household cigarettes smoked, percent of ETS exposure during lifetime, and the number of current smokers at home all showed increasing trends in the risks of respiratory outcomes, consistent with the dose-response relationship of household ETS in many recent studies [34,37,38]. In a recent meta-analysis of the effects of household ETS on asthma and wheeze, Vork et al reported a summary relative risk for asthma of 1.21 (95% CI 1.17-1.26) that is consistent with our estimate of 1.15 (95% CI 0.92-1.44). Our estimate for the association between household ETS and active asthma (1.39, 95% CI 1.00-1.93) is slightly higher than that from the meta-analysis (1.25, 95% CI 1.21-1.30), but the confidence intervals show considerable overlap [39]. The literature on the relationship between household ETS exposure and respiratory symptoms gives conflicting results with regard to sex differences in susceptibility [40]. We found almost all respiratory outcomes showed non-significant interaction between household ETS exposure and sex in health outcomes (Table S4 in Additional file 1), consistent with the findings from a recent study in Singapore [38].

The prevalence of maternal and paternal smoking in this study was 3.4% and 36.0%, respectively, which is comparable to an earlier survey in Singapore [41]. The prevalence of maternal smoking in Taiwan is much lower than the 13% reported in Sweden [42], 23.8% in USA [43], and 32% in Austria [44]. Studies using cotinine as a biomarker show that the strength of the association between maternal smoking and respiratory outcomes is in part due to larger ETS doses from maternal smoking than from other sources [24]. In our study, maternal ETS conferred a higher risk of respiratory symptoms compared with paternal ETS (Table 3 and Table 4). Several reasons could explain this phenomenon: mothers have more direct contact with their children at home compared with fathers; women who smoke during pregnancy are likely to continue smoking after delivery. In Taiwan, the ratio of current smoker/ex-smoker rates in adulthood is close to 7 [15], far higher than the ratio near to one in the United States [45] and indicating a particularly low rate of smoking cessation in Taiwanese adults. Depending on which symptom is considered, our results show the higher risk for ETS from both parents when compared with just maternal or just paternal ETS exposure (Table 5). The reasons for this are not clear and could be partly attributed to the behavior of the parents [23].

We found that *in utero *exposure to maternal smoking had larger effects on early-onset asthma than those asthmatics diagnosed after 5 years of age (Table 6). Previous studies showed a stronger risk for incident asthma or wheezing illness among younger children compared with older children [21,31]. These investigators suggested that the stronger relationship might be attributed to exacerbation of intercurrent infection among young children, resulting in transient wheeze that would tend to diminish with age and increasing airway caliber. The proposed mechanism would suggest that household ETS may not be the sole primary cause of early childhood asthma.

Our study has some limitations. Because of cross-sectional data, the factors we studied may have affected outcome prevalence through effects on disease duration rather than disease incidence. Biases could also be introduced if parents or children change their time-activity patterns to avoid ETS exposure. However, we note that the prevalence of past-only ETS exposure is very low, suggesting that adult smoking patterns would not differentially change over time. Differential participation by children with respiratory outcomes who had different ETS exposure histories is unlikely to have been significant enough to produce substantial bias, as participation rates in each classroom were high. Retrospective recall of tobacco smoking by questionnaire is likely to have produced some misclassification of exposure. However, the validity of ETS exposure estimates based on questionnaire responses have been investigated and found to provide reasonably valid data [46-49]. It can be expected that more parents would not want to be seen as flouting the law and thus report that they are smoking within the privacy of their homes. Under these conditions, it can be anticipated that the proportions of exposure misclassification are likely to be non-differential for symptomatic children as for the healthy children.

In summary, our results showed that prenatal and current household ETS exposure in Taiwan had significant adverse effects on respiratory health in children. Eliminating household ETS exposure may offer the most promising opportunity for reducing morbidity, because this risk factor is potentially modifiable. Public health policy for reducing the burden of respiratory symptoms may require a stronger focus on smoking cessation in the home, where children could gain significant health benefits.

## Competing interests

The authors declare that they have no competing interests.

## Authors' contributions

CHT analyzed data and drafted this paper. JHH was involved in the study design and field work. BFH was involved with statistical conception and critical revision of the manuscript. YLL was the coordinator of TCHS, who worked on content development, statistical analysis, obtaining funding, and supervision of the study.

## Supplementary Material

Additional file 1 **Table S1. **Characteristics of the study participants in TCHS by sex. Table S2. Demographic characteristics for the percentage of household environmental tobacco smoke (ETS) exposure categories in TCHS. Table S3. Percentage of participants in TCHS with asthma, wheeze and bronchitic symptoms within household environmental tobacco smoke (ETS) exposure categories. Table S4. Effects of household environmental tobacco smoke (ETS) exposure on subcategories of asthma, wheeze and bronchitic symptoms, stratified by sex. Click here for file
