# Supplementary material for: Household environmental tobacco smoke and risks of asthma, wheeze and bronchitic symptoms among children in Taiwan
Source: Respir Res. 2010 Jan 29;11(1):11. doi: 10.1186/1465-9921-11-11 (PMC2828425; doi:10.1186/1465-9921-11-11)
Supplement: Additional file 1 — Table S1. Characteristics of the study participants in TCHS by sex. Table S2. Demographic characteristics for the percentage of household environmental tobacco smoke (ETS) exposure categories in TCHS. Table S3. Percentage of participants in TCHS with asthma, wheeze and bronchitic symptoms within household environmental tobacco smoke (ETS) exposure categories. Table S4. Effects of household environmental tobacco smoke (ETS) exposure on subcategories of asthma, wheeze and bronchitic symptoms, stratified by sex. [file 1465-9921-11-11-S1.DOC]

| **Table S1. Characteristics of the study participants in TCHS by sex** | | | | | | |
| --- | --- | --- | --- | --- | --- | --- |
|  | Total | | Boys | | Girls | |
|  | (n=5019) | | (n=2432) | | (n=2587) | |
|  | n | % | n | % | n | % |
| Home environmental factors |  |  |  |  |  |  |
| Dog | 1460 | 29.4 | 671 | 27.9 | 789 | 30.8 |
| Cat | 274 | 5.5 | 122 | 5.1 | 152 | 5.9 |
| Other pets | 1454 | 29.4 | 659 | 27.4 | 795 | 31.2 |
| Incense burning | 3159 | 63.6 | 1523 | 63.4 | 1636 | 63.8 |
| Carpet use | 432 | 8.7 | 204 | 8.4 | 228 | 8.9 |
| Cockroaches | 4259 | 85.7 | 2034 | 84.4 | 2225 | 87.0 |
| Visible mould | 1731 | 34.8 | 847 | 35.1 | 884 | 34.5 |
| Water damage | 558 | 11.2 | 253 | 10.5 | 305 | 11.9 |
| Mildewy odor | 756 | 15.1 | 345 | 14.3 | 411 | 16.0 |
| Water stamp on the wall | 1316 | 26.4 | 608 | 25.2 | 708 | 27.6 |
| Air cleaner use | 822 | 16.4 | 390 | 16.1 | 432 | 16.7 |
| Air conditioner use | 4597 | 92.3 | 2207 | 91.7 | 2390 | 92.8 |
| Dehumidifier use | 2087 | 41.7 | 1009 | 41.6 | 1078 | 41.7 |
| Number of full siblings |  |  |  |  |  |  |
| 0 | 468 | 9.3 | 241 | 9.9 | 227 | 8.8 |
| 1 | 2245 | 44.7 | 1167 | 48.0 | 1078 | 41.7 |
| 2 | 1691 | 33.7 | 783 | 32.2 | 908 | 35.1 |
| ≧3 | 615 | 12.3 | 241 | 9.9 | 374 | 14.5 |
| Low birth weight | 611 | 13.0 | 284 | 12.5 | 327 | 13.4 |
| Neonatal special care | 427 | 8.6 | 231 | 9.6 | 196 | 7.7 |
| Neonatal oxygen use |  |  |  |  |  |  |
| 0 | 4518 | 91.2 | 2160 | 90.3 | 2358 | 92.1 |
| <2 wk | 137 | 2.8 | 75 | 3.1 | 62 | 2.4 |
| 2~4 wk | 34 | 0.7 | 22 | 0.9 | 12 | 0.5 |
| ≧4 wk | 22 | 0.4 | 10 | 0.4 | 12 | 0.5 |
| Oxygen use at home | 31 | 0.6 | 17 | 0.7 | 14 | 0.5 |
| Hay fever | 1287 | 25.9 | 741 | 30.8 | 546 | 21.3 |
| Severe chest illness before age 2 |  |  |  |  |  |  |
| 0 | 4632 | 92.7 | 2202 | 91.0 | 2430 | 94.3 |
| 1 times | 199 | 4.0 | 120 | 5.0 | 79 | 3.1 |
| ≧ 2 times | 165 | 3.3 | 97 | 4.0 | 68 | 2.6 |
| Severe chest illness after age 2 |  |  |  |  |  |  |
| 0 | 4505 | 90.6 | 2153 | 89.6 | 2352 | 91.6 |
| 1 times | 209 | 4.2 | 108 | 4.5 | 101 | 3.9 |
| ≧ 2 times | 257 | 5.2 | 142 | 5.9 | 115 | 4.5 |
| Outdoor exercise | 3365 | 68.5 | 1824 | 76.9 | 1541 | 60.6 |
| Time of outdoor exercise |  |  |  |  |  |  |
| 0 | 1547 | 31.9 | 547 | 23.5 | 1000 | 39.7 |
| <1 hr | 1589 | 32.8 | 734 | 31.6 | 855 | 33.9 |
| ≧1 hr | 1710 | 35.3 | 1045 | 44.9 | 665 | 26.4 |
| Number of subjects does not add up to total N because of missing data. | | | | | | |

**Table S2. Demographic characteristics for the percentage of household environmental tobacco smoke (ETS) exposure categories in TCHS**

|  | *In utero*  exposure | |  | Lifetime  ETS | |  | Currently  ETS | |  | Dad  smoking | |  | Mom  smoking | |  | Others  smoking | |
| --- | --- | --- | --- | --- | --- | --- | --- | --- | --- | --- | --- | --- | --- | --- | --- | --- | --- |
|  | Yes | % |  | Yes | % |  | Yes | % |  | Yes | % |  | Yes | % |  | Yes | % |
| Sex |  |  |  |  |  |  |  |  |  |  |  |  |  |  |  |  |  |
| Boys | 78 | 3.3 |  | 1155 | 48.4 |  | 1061 | 44.4 |  | 825 | 34.6 |  | 63 | 2.6 |  | 343 | 14.4 |
| Girls | 114 | 4.4 |  | 1266 | 49.4 |  | 1161 | 45.3 |  | 955 | 37.2 |  | 101 | 3.9 |  | 433 | 16.9 |
|  |  |  |  |  |  |  |  |  |  |  |  |  |  |  |  |  |  |
| Parental education, yr |  |  |  |  |  |  |  |  |  |  |  |  |  |  |  |  |  |
| ≦12 | 154 | 4.9 |  | 1765 | 56.4 |  | 1642 | 52.5 |  | 1339 | 42.9 |  | 133 | 4.3 |  | 551 | 17.7 |
| 13~15 | 22 | 2.3 |  | 384 | 41.1 |  | 349 | 37.4 |  | 276 | 29.4 |  | 14 | 1.5 |  | 120 | 12.8 |
| ≧16 | 15 | 1.8 |  | 256 | 30.1 |  | 218 | 25.6 |  | 153 | 17.8 |  | 16 | 1.9 |  | 98 | 11.4 |
|  |  |  |  |  |  |  |  |  |  |  |  |  |  |  |  |  |  |
| Family income* |  |  |  |  |  |  |  |  |  |  |  |  |  |  |  |  |  |
| ≦400,000 | 101 | 5.9 |  | 936 | 54.5 |  | 872 | 50.8 |  | 666 | 38.8 |  | 75 | 4.4 |  | 351 | 20.5 |
| 410,000~800,000 | 56 | 3.1 |  | 930 | 51.2 |  | 846 | 46.6 |  | 722 | 39.8 |  | 63 | 3.5 |  | 252 | 13.9 |
| ≧810,000 | 17 | 1.6 |  | 370 | 35.4 |  | 331 | 31.6 |  | 282 | 26.9 |  | 12 | 1.1 |  | 99 | 9.4 |
|  |  |  |  |  |  |  |  |  |  |  |  |  |  |  |  |  |  |
| Number of siblings |  |  |  |  |  |  |  |  |  |  |  |  |  |  |  |  |  |
| 0 | 48 | 10.4 |  | 242 | 52.3 |  | 222 | 47.9 |  | 123 | 26.6 |  | 24 | 5.2 |  | 99 | 21.4 |
| 1 | 68 | 3.1 |  | 1039 | 46.8 |  | 946 | 42.6 |  | 763 | 34.3 |  | 66 | 3.0 |  | 314 | 14.1 |
| 2 | 58 | 3.5 |  | 809 | 48.6 |  | 746 | 44.8 |  | 624 | 37.5 |  | 50 | 3.0 |  | 258 | 15.5 |
| ≧3 | 18 | 3.0 |  | 331 | 54.9 |  | 308 | 51.1 |  | 270 | 44.6 |  | 24 | 4.0 |  | 105 | 17.4 |
| *New Taiwan dollars per year ($1 US = $ 33 New Taiwan). | | | | | | | | | | | | | | | | | |
| Number of subjects does not add up to total N because of missing data. | | | | | | | | | | | | | | | | | |

| **Table S3. Percentage of participants in TCHS with asthma, wheeze and bronchitic symptoms within household environmental tobacco smoke (ETS) exposure categories** | | | | | | | | | | | | | | | | | |
| --- | --- | --- | --- | --- | --- | --- | --- | --- | --- | --- | --- | --- | --- | --- | --- | --- | --- |
|  | *In utero*  exposure | |  | Lifetime  ETS | |  | Currently  ETS | |  | Dad  smoking | |  | Mom  smoking | |  | Others  smoking | |
|  | Yes | No |  | Yes | No |  | Yes | No |  | Yes | No |  | Yes | No |  | Yes | No |
|  | 192 | 4749 |  | 2414 | 2520 |  | 2215 | 2719 |  | 1774 | 3165 |  | 162 | 4777 |  | 776 | 4163 |
| Asthma |  |  |  |  |  |  |  |  |  |  |  |  |  |  |  |  |  |
| Ever asthma | 11.6 | 7.3 |  | 7.7 | 7.2 |  | 7.9 | 7.1 |  | 7.5 | 7.4 |  | 10.4 | 7.4 |  | 7.7 | 7.4 |
| Active asthma | 7.9 | 3.2 |  | 3.9 | 2.9 |  | 4.0 | 2.9 |  | 3.6 | 3.3 |  | 6.1 | 3.3 |  | 4.5 | 3.2 |
| Early-onset asthma* | 7.7 | 4.8 |  | 4.9 | 4.9 |  | 5.0 | 4.8 |  | 4.7 | 5.0 |  | 7.5 | 4.8 |  | 4.4 | 5.0 |
| Late-onset asthma** | 4.5 | 2.5 |  | 2.7 | 2.4 |  | 2.8 | 2.4 |  | 2.9 | 2.4 |  | 2.6 | 2.6 |  | 3.4 | 2.4 |
| Treatments for asthma |  |  |  |  |  |  |  |  |  |  |  |  |  |  |  |  |  |
| Medication use | 5.8 | 2.3 |  | 2.8 | 2.2 |  | 3.0 | 2.1 |  | 2.6 | 2.4 |  | 3.0 | 2.4 |  | 3.6 | 2.2 |
| ER visit or hospitalization | 5.6 | 1.1 |  | 1.6 | 1.0 |  | 1.7 | 0.9 |  | 1.5 | 1.1 |  | 3.9 | 1.2 |  | 2.6 | 1.0 |
| Wheeze |  |  |  |  |  |  |  |  |  |  |  |  |  |  |  |  |  |
| Ever wheeze | 20.3 | 11.3 |  | 12.8 | 10.5 |  | 12.8 | 10.6 |  | 11.7 | 11.5 |  | 18.5 | 11.4 |  | 14.4 | 11.1 |
| Current wheeze | 11.6 | 3.4 |  | 4.3 | 3.1 |  | 4.2 | 3.3 |  | 3.6 | 3.7 |  | 9.9 | 3.5 |  | 5.1 | 3.4 |
| Awakened at night | 6.8 | 2.0 |  | 2.6 | 1.7 |  | 2.8 | 1.6 |  | 2.7 | 1.9 |  | 4.9 | 2.1 |  | 3.6 | 1.9 |
| Bronchitic symptoms |  |  |  |  |  |  |  |  |  |  |  |  |  |  |  |  |  |
| Bronchitis | 9.4 | 5.6 |  | 6.7 | 4.8 |  | 6.6 | 5.0 |  | 5.6 | 5.8 |  | 8.6 | 5.6 |  | 7.2 | 5.4 |
| Chronic cough | 7.4 | 3.2 |  | 3.8 | 2.9 |  | 3.7 | 3.0 |  | 3.3 | 3.4 |  | 8.0 | 3.2 |  | 4.1 | 3.2 |
| Phlegm without cold | 9.5 | 4.1 |  | 4.6 | 4.0 |  | 4.5 | 4.2 |  | 4.9 | 4.1 |  | 7.9 | 4.2 |  | 5.0 | 4.3 |
| *Early-onset: asthma diagnosed ≦5 yr of age. | | | | | | | | | | | | | | | | | |
| **Late-onset: asthma diagnosed >5 yr of age. | | | | | | | | | | | | | | | | | |

| **Table S4. Effects of household environmental tobacco smoke (ETS) exposure on subcategories of asthma, wheeze and bronchitic symptoms, stratified by sex** | | | | | | | | | | | | | | | |
| --- | --- | --- | --- | --- | --- | --- | --- | --- | --- | --- | --- | --- | --- | --- | --- |
|  | Asthma | | | | | | | | | | | | | | |
|  | Ever asthma | | | | | | |  | Active asthma | | | | | | |
|  | Boy* | |  | Girl* | |  | p value** |  | Boy* | |  | Girl* | |  | p value** |
|  | OR | 95%CI |  | OR | 95%CI |  |  |  | OR | 95%CI |  | OR | 95%CI |  |  |
| ETS |  |  |  |  |  |  |  |  |  |  |  |  |  |  |  |
| *In utero* exposure | 1.20 | (0.56,2.59) |  | 1.69 | (0.90,3.18) |  | 0.60 |  | 1.15 | (0.42,3.17) |  | 2.77 | (1.33,5.80) |  | 0.28 |
| Currently | 1.14 | (0.83,1.56) |  | 1.15 | (0.83,1.60) |  | 0.63 |  | 1.27 | (0.80,2.00) |  | 1.54 | (0.96,2.48) |  | 0.47 |
| Previous only | 0.60 | (0.24,1.55) |  | 0.97 | (0.43,2.16) |  | 0.49 |  | 0.24 | (0.03,1.80) |  | 1.20 | (0.42,3.41) |  | 0.12 |
| ETS sources |  |  |  |  |  |  |  |  |  |  |  |  |  |  |  |
| Dad | 0.90 | (0.64,1.26) |  | 1.26 | (0.91,1.76) |  | 0.08 |  | 0.84 | (0.51,1.37) |  | 1.44 | (0.89,2.32) |  | 0.11 |
| Mom | 1.90 | (0.88,4.12) |  | 1.04 | (0.48,2.22) |  | 0.24 |  | 2.37 | (0.92,6.10) |  | 1.14 | (0.40,3.28) |  | 0.25 |
| Number of smokers |  |  |  |  |  |  |  |  |  |  |  |  |  |  |  |
| 0 | 1 |  |  | 1 |  |  | 0.30 |  | 1 |  |  | 1 |  |  | 0.11 |
| 1 | 0.81 | (0.56,1.18) |  | 1.02 | (0.70,1.48) |  |  |  | 0.86 | (0.51,1.47) |  | 1.24 | (0.73,2.13) |  |  |
| 2 | 0.58 | (0.30,1.11) |  | 1.07 | (0.62,1.85) |  |  |  | 0.51 | (0.18,1.45) |  | 1.39 | (0.66,2.91) |  |  |
| ≧3 | 1.68 | (0.88,3.18) |  | 1.60 | (0.87,2.93) |  |  |  | 3.03 | (1.42,6.50) |  | 1.74 | (0.72,4.18) |  |  |
| p value for trend | 0.87 |  |  | 0.24 |  |  |  |  | 0.22 |  |  | 0.16 |  |  |  |
| Currently amount of ETS*** |  |  |  |  |  |  |  |  |  |  |  |  |  |  |  |
| 0 | 1 |  |  | 1 |  |  | 0.45 |  | 1 |  |  | 1 |  |  | 0.66 |
| ≦10 | 1.01 | (0.71,1.43) |  | 1.11 | (0.78,1.57) |  |  |  | 1.11 | (0.67,1.85) |  | 1.42 | (0.85,2.39) |  |  |
| > 10 | 1.65 | (1.00,2.73) |  | 1.25 | (0.74,2.10) |  |  |  | 2.13 | (1.07,4.22) |  | 2.10 | (1.07,4.10) |  |  |
| p value for trend | 0.14 |  |  | 0.37 |  |  |  |  | 0.07 |  |  | 0.03 |  |  |  |
| Percent of ETS# |  |  |  |  |  |  |  |  |  |  |  |  |  |  |  |
| 0 | 1 |  |  | 1 |  |  | 0.89 |  | 1 |  |  | 1 |  |  | 0.74 |
| ≦ 20% | 0.96 | (0.66,1.39) |  | 1.00 | (0.68,1.47) |  |  |  | 1.07 | (0.63,1.83) |  | 1.18 | (0.66,2.09) |  |  |
| > 20% | 1.49 | (0.98,2.28) |  | 1.39 | (0.92,2.10) |  |  |  | 1.58 | (0.86,2.89) |  | 2.08 | (1.18,3.67) |  |  |
| p value for trend | 0.13 |  |  | 0.17 |  |  |  |  | 0.18 |  |  | 0.02 |  |  |  |
| *Models are adjusted for age, parental education, family history of asthma, family history of atopy, gestational age, and community. | | | | | | | | | | | | | | | |
| **Test for interaction between sex and environmental tobacco smoke exposure. | | | | | | | | | | | | | | | |
| ***Average cigarettes per day | | | | | | | | | | | | | | | |
| # Average percent of ETS in lifetime | | | | | | | | | | | | | | | |

| **Table S4. Effects of household environmental tobacco smoke (ETS) exposure on subcategories of asthma, wheeze and bronchitic symptoms, stratified by sex (cont)** | | | | | | | | | | | | | | | |
| --- | --- | --- | --- | --- | --- | --- | --- | --- | --- | --- | --- | --- | --- | --- | --- |
|  | Treatments for asthma | | | | | | | | | | | | | | |
|  | Medication use | | | | | | |  | ER visit or hospitalization | | | | | | |
|  | Boy* | |  | Girl* | |  | p value** |  | Boy* | |  | Girl* | |  | p value** |
|  | OR | 95%CI |  | OR | 95%CI |  |  |  | OR | 95%CI |  | OR | 95%CI |  |  |
| ETS |  |  |  |  |  |  |  |  |  |  |  |  |  |  |  |
| *In utero* exposure | 1.66 | (0.59,4.69) |  | 2.01 | (0.80,5.06) |  | 0.99 |  | 3.92 | (1.15,13.40) |  | 5.62 | (2.05,15.40) |  | 0.71 |
| Currently | 1.02 | (0.60,1.74) |  | 1.75 | (1.00,3.06) |  | 0.12 |  | 1.50 | (0.64,3.51) |  | 1.79 | (0.85,3.75) |  | 0.92 |
| Previous only | 0.33 | (0.04,2.46) |  | 0.77 | (0.18,3.30) |  | 0.49 |  | NA |  |  | NA |  |  |  |
| ETS sources |  |  |  |  |  |  |  |  |  |  |  |  |  |  |  |
| Dad | 0.73 | (0.41,1.31) |  | 1.27 | (0.73,2.23) |  | 0.18 |  | 0.91 | (0.37,2.19) |  | 1.53 | (0.74,3.14) |  | 0.58 |
| Mom | 1.37 | (0.39,4.82) |  | 0.64 | (0.15,2.77) |  | 0.39 |  | 7.12 | (2.09,24.30) |  | 1.59 | (0.36,7.04) |  | 0.07 |
| Number of smokers |  |  |  |  |  |  |  |  |  |  |  |  |  |  |  |
| 0 | 1 |  |  | 1 |  |  | 0.12 |  | 1 |  |  | 1 |  |  | 0.06 |
| 1 | 0.68 | (0.36,1.29) |  | 1.37 | (0.74,2.56) |  |  |  | 0.37 | (0.10,1.36) |  | 1.63 | (0.72,3.71) |  |  |
| 2 | 0.46 | (0.14,1.56) |  | 1.08 | (0.43,2.74) |  |  |  | 0.35 | (0.04,2.79) |  | 0.80 | (0.17,3.70) |  |  |
| ≧3 | 3.45 | (1.54,7.74) |  | 2.01 | (0.76,5.31) |  |  |  | 6.90 | (2.46,19.30) |  | 3.11 | (1.01,9.57) |  |  |
| p value for trend | 0.19 |  |  | 0.23 |  |  |  |  | 0.01 |  |  | 0.13 |  |  |  |
| Currently amount of ETS*** |  |  |  |  |  |  |  |  |  |  |  |  |  |  |  |
| 0 | 1 |  |  | 1 |  |  | 0.13 |  | 1 |  |  | 1 |  |  | 0.89 |
| ≦10 | 0.77 | (0.42,1.43) |  | 1.63 | (0.89,2.98) |  |  |  | 1.23 | (0.48,3.15) |  | 1.17 | (0.50,2.73) |  |  |
| > 10 | 2.32 | (1.12,4.78) |  | 2.32 | (1.07,5.05) |  |  |  | 2.77 | (0.92,8.38) |  | 2.69 | (1.00,7.23) |  |  |
| p value for trend | 0.15 |  |  | 0.02 |  |  |  |  | 0.11 |  |  | 0.09 |  |  |  |
| Percent of ETS# |  |  |  |  |  |  |  |  |  |  |  |  |  |  |  |
| 0 | 1 |  |  | 1 |  |  | 0.32 |  | 1 |  |  | 1 |  |  | 0.91 |
| ≦ 20% | 0.91 | (0.49,1.69) |  | 1.54 | (0.81,2.96) |  |  |  | 1.08 | (0.39,2.97) |  | 1.06 | (0.43,2.64) |  |  |
| > 20% | 1.20 | (0.59,2.46) |  | 1.95 | (0.99,3.86) |  |  |  | 2.00 | (0.70,5.73) |  | 2.69 | (1.17,6.17) |  |  |
| p value for trend | 0.74 |  |  | 0.05 |  |  |  |  | 0.24 |  |  | 0.03 |  |  |  |

| **Table S4. Effects of household environmental tobacco smoke (ETS) exposure on subcategories of asthma, wheeze and bronchitic symptoms, stratified by sex (cont)** | | | | | | | | | | | | | | | |
| --- | --- | --- | --- | --- | --- | --- | --- | --- | --- | --- | --- | --- | --- | --- | --- |
|  | Wheeze | | | | | | | | | | | | | | |
|  | Ever wheeze | | | | | | |  | Current wheeze | | | | | | |
|  | Boy* | |  | Girl* | |  | p value** |  | Boy* | |  | Girl* | |  | p value** |
|  | OR | 95%CI |  | OR | 95%CI |  |  |  | OR | 95%CI |  | OR | 95%CI |  |  |
| ETS |  |  |  |  |  |  |  |  |  |  |  |  |  |  |  |
| *In utero* exposure | 1.42 | (0.76,2.66) |  | 2.41 | (1.49,3.91) |  | 0.14 |  | 1.92 | (0.80,4.64) |  | 3.91 | (2.09,7.30) |  | 0.21 |
| Currently | 1.21 | (0.94,1.57) |  | 1.37 | (1.05,1.78) |  | 0.28 |  | 1.16 | (0.74,1.83) |  | 1.44 | (0.93,2.22) |  | 0.55 |
| Previous only | 0.88 | (0.45,1.76) |  | 1.38 | (0.77,2.50) |  | 0.28 |  | 0.29 | (0.04,2.17) |  | 2.65 | (1.27,5.53) |  | 0.01 |
| ETS sources |  |  |  |  |  |  |  |  |  |  |  |  |  |  |  |
| Dad | 0.84 | (0.63,1.11) |  | 1.42 | (1.09,1.86) |  | 0.002 |  | 0.75 | (0.46,1.23) |  | 1.20 | (0.77,1.86) |  | 0.18 |
| Mom | 1.97 | (1.02,3.81) |  | 1.61 | (0.92,2.81) |  | 0.75 |  | 2.85 | (1.17,6.93) |  | 2.58 | (1.22,5.46) |  | 0.87 |
| Number of smokers |  |  |  |  |  |  |  |  |  |  |  |  |  |  |  |
| 0 | 1 |  |  | 1 |  |  | 0.004 |  | 1 |  |  | 1 |  |  | 0.06 |
| 1 | 0.85 | (0.63,1.15) |  | 1.15 | (0.85,1.57) |  |  |  | 0.89 | (0.53,1.51) |  | 1.03 | (0.62,1.73) |  |  |
| 2 | 0.86 | (0.54,1.37) |  | 2.20 | (1.50,3.23) |  |  |  | 0.77 | (0.34,1.78) |  | 2.22 | (1.24,3.98) |  |  |
| ≧3 | 1.79 | (1.04,3.07) |  | 1.57 | (0.92,2.67) |  |  |  | 1.98 | (0.86,4.58) |  | 1.00 | (0.37,2.66) |  |  |
| p value for trend | 0.48 |  |  | 0.001 |  |  |  |  | 0.53 |  |  | 0.13 |  |  |  |
| Currently amount of ETS*** |  |  |  |  |  |  |  |  |  |  |  |  |  |  |  |
| 0 | 1 |  |  | 1 |  |  | 0.46 |  | 1 |  |  | 1 |  |  | 0.68 |
| ≦10 | 1.12 | (0.84,1.48) |  | 1.3 | (0.97,1.72) |  |  |  | 1.13 | (0.69,1.85) |  | 1.48 | (0.93,2.35) |  |  |
| > 10 | 1.68 | (1.10,2.55) |  | 1.62 | (1.07,2.44) |  |  |  | 1.36 | (0.65,2.82) |  | 1.47 | (0.74,2.90) |  |  |
| p value for trend | 0.03 |  |  | 0.01 |  |  |  |  | 0.40 |  |  | 0.12 |  |  |  |
| Percent of ETS# |  |  |  |  |  |  |  |  |  |  |  |  |  |  |  |
| 0 | 1 |  |  | 1 |  |  | 0.57 |  | 1 |  |  | 1 |  |  | 0.91 |
| ≦ 20% | 1.12 | (0.83,1.51) |  | 1.19 | (0.87,1.62) |  |  |  | 0.87 | (0.50,1.52) |  | 1.01 | (0.59,1.74) |  |  |
| > 20% | 1.45 | (1.02,2.07) |  | 1.66 | (1.18,2.32) |  |  |  | 1.89 | (1.08,3.29) |  | 2.24 | (1.35,3.72) |  |  |
| p value for trend | 0.046 |  |  | 0.004 |  |  |  |  | 0.07 |  |  | 0.005 |  |  |  |

| **Table S4. Effects of household environmental tobacco smoke (ETS) exposure on subcategories of asthma, wheeze and bronchitic symptoms, stratified by sex (cont)** | | | | | | | | | | | | | | | |
| --- | --- | --- | --- | --- | --- | --- | --- | --- | --- | --- | --- | --- | --- | --- | --- |
|  | Attacks of wheeze | | | | | | |  | Bronchitic symptoms | | | | | | |
|  | Awakened at night | | | | | | |  | Brochitis | | | | | | |
|  | Boy* | |  | Girl* | |  | p value** |  | Boy* | |  | Girl* | |  | p value** |
|  | OR | 95%CI |  | OR | 95%CI |  |  |  | OR | 95%CI |  | OR | 95%CI |  |  |
| ETS |  |  |  |  |  |  |  |  |  |  |  |  |  |  |  |
| *In utero* exposure | 1.91 | (0.53,6.86) |  | 3.64 | (1.73,7.64) |  | 0.37 |  | 2.12 | (1.01,4.45) |  | 1.56 | (0.72,3.39) |  | 0.46 |
| Currently | 1.30 | (0.67,2.52) |  | 1.91 | (1.13,3.24) |  | 0.37 |  | 1.39 | (0.98,1.96) |  | 1.34 | (0.92,1.96) |  | 0.87 |
| Previous only | NA | NA |  | 0.34 | (0.05,2.53) |  |  |  | 1.55 | (0.71,3.37) |  | 1.45 | (0.65,3.24) |  | 0.98 |
| ETS sources |  |  |  |  |  |  |  |  |  |  |  |  |  |  |  |
| Dad | 1.15 | (0.58,2.29) |  | 1.59 | (0.95,2.64) |  | 0.41 |  | 0.90 | (0.62,1.30) |  | 1.14 | (0.77,1.68) |  | 0.41 |
| Mom | 4.40 | (1.21,16.00) |  | 1.73 | (0.66,4.49) |  | 0.55 |  | 2.31 | (1.02,5.21) |  | 1.15 | (0.48,2.78) |  | 0.30 |
| Number of smokers |  |  |  |  |  |  |  |  |  |  |  |  |  |  |  |
| 0 | 1 |  |  | 1 |  |  | 0.66 |  | 1 |  |  | 1 |  |  | 0.01 |
| 1 | 1.09 | (0.50,2.37) |  | 1.62 | (0.88,2.95) |  |  |  | 0.68 | (0.44,1.04) |  | 1.36 | (0.90,2.07) |  |  |
| 2 | 1.24 | (0.40,3.80) |  | 2.15 | (1.02,4.55) |  |  |  | 1.29 | (0.75,2.24) |  | 1.30 | (0.71,2.36) |  |  |
| ≧3 | 3.96 | (1.41,11.10) |  | 3.21 | (1.41,7.32) |  |  |  | 2.48 | (1.28,4.82) |  | 0.83 | (0.32,2.16) |  |  |
| p value for trend | 0.04 |  |  | 0.002 |  |  |  |  | 0.07 |  |  | 0.57 |  |  |  |
| Currently amount of ETS*** |  |  |  |  |  |  |  |  |  |  |  |  |  |  |  |
| 0 | 1 |  |  | 1 |  |  | 0.22 |  | 1 |  |  | 1 |  |  | 0.90 |
| ≦10 | 1.32 | (0.65,2.66) |  | 1.45 | (0.81,2.58) |  |  |  | 1.29 | (0.89,1.88) |  | 1.31 | (0.87,1.96) |  |  |
| > 10 | 1.12 | (0.35,3.65) |  | 3.39 | (1.74,6.60) |  |  |  | 1.64 | (0.92,2.92) |  | 1.36 | (0.74,2.48) |  |  |
| p value for trend | 0.60 |  |  | 0.001 |  |  |  |  | 0.06 |  |  | 0.18 |  |  |  |
| Percent of ETS# |  |  |  |  |  |  |  |  |  |  |  |  |  |  |  |
| 0 | 1 |  |  | 1 |  |  | 0.37 |  |  |  |  |  |  |  | 0.71 |
| ≦ 20% | 1.22 | (0.57,2.60) |  | 1.09 | (0.57,2.12) |  |  |  | 1.16 | (0.78,1.74) |  | 1.27 | (0.82,1.95) |  |  |
| > 20% | 1.59 | (0.67,3.74) |  | 2.93 | (1.63,5.26) |  |  |  | 1.83 | (1.16,2.89) |  | 1.46 | (0.90,2.37) |  |  |
| p value for trend | 0.29 |  |  | 0.001 |  |  |  |  | 0.02 |  |  | 0.11 |  |  |  |

| **Table S4. Effects of household environmental tobacco smoke (ETS) exposure on subcategories of asthma, wheeze and bronchitic symptoms, stratified by sex (cont)** | | | | | | | | | | | | | | | |
| --- | --- | --- | --- | --- | --- | --- | --- | --- | --- | --- | --- | --- | --- | --- | --- |
|  | Bronchitic symptoms | | | | | | | | | | | | | | |
|  | Chronic cough | | | | | | |  | Phlegm without cold | | | | | | |
|  | Boy* | |  | Girl* | |  | p value** |  | Boy* | |  | Girl* | |  | p value** |
|  | OR | 95%CI |  | OR | 95%CI |  |  |  | OR | 95%CI |  | OR | 95%CI |  |  |
| ETS |  |  |  |  |  |  |  |  |  |  |  |  |  |  |  |
| *In utero* exposure | 2.66 | (1.21,5.85) |  | 1.18 | (0.45,3.09) |  | 0.14 |  | 1.35 | (0.58,3.17) |  | 2.44 | (1.24,4.82) |  | 0.48 |
| Currently | 1.39 | (0.89,2.18) |  | 0.90 | (0.56,1.44) |  | 0.18 |  | 0.82 | (0.54,1.23) |  | 1.20 | (0.80,1.81) |  | 0.13 |
| Previous only | 0.81 | (0.25,2.65) |  | 2.52 | (1.10,5.77) |  | 0.11 |  | 2.12 | (0.97,4.65) |  | 1.19 | (0.47,3.03) |  | 0.40 |
| ETS sources |  |  |  |  |  |  |  |  |  |  |  |  |  |  |  |
| Dad | 1.16 | (0.74,1.83) |  | 0.81 | (0.49,1.34) |  | 0.32 |  | 1.28 | (0.85,1.93) |  | 1.13 | (0.75,1.72) |  | 0.32 |
| Mom | 3.88 | (1.70,8.86) |  | 1.36 | (0.52,3.52) |  | 0.12 |  | 1.56 | (0.58,4.19) |  | 1.89 | (0.87,4.09) |  | 0.12 |
| Number of smokers |  |  |  |  |  |  |  |  |  |  |  |  |  |  |  |
| 0 | 1 |  |  | 1 |  |  | 0.25 |  | 1 |  |  | 1 |  |  | 0.74 |
| 1 | 0.87 | (0.50,1.51) |  | 0.73 | (0.42,1.28) |  |  |  | 1.27 | (0.81,2.01) |  | 0.82 | (0.50,1.34) |  |  |
| 2 | 1.52 | (0.75,3.05) |  | 0.59 | (0.24,1.41) |  |  |  | 1.25 | (0.63,2.47) |  | 1.04 | (0.54,2.01) |  |  |
| ≧3 | 2.75 | (1.27,5.93) |  | 1.17 | (0.47,2.92) |  |  |  | 2.56 | (1.23,5.34) |  | 2.38 | (1.22,4.64) |  |  |
| p value for trend | 0.03 |  |  | 0.52 |  |  |  |  | 0.03 |  |  | 0.09 |  |  |  |
| Currently amount of ETS*** |  |  |  |  |  |  |  |  |  |  |  |  |  |  |  |
| 0 | 1 |  |  | 1 |  |  | 0.22 |  | 1 |  |  | 1 |  |  | 0.28 |
| ≦10 | 1.20 | (0.73,1.96) |  | 0.59 | (0.33,1.06) |  |  |  | 0.75 | (0.48,1.18) |  | 1.10 | (0.70,1.72) |  |  |
| > 10 | 2.44 | (1.29,4.63) |  | 2.09 | (1.13,3.87) |  |  |  | 1.02 | (0.53,1.96) |  | 1.74 | (0.95,3.16) |  |  |
| p value for trend | 0.02 |  |  | 0.26 |  |  |  |  | 0.58 |  |  | 0.12 |  |  |  |
| Percent of ETS# |  |  |  |  |  |  |  |  |  |  |  |  |  |  |  |
| 0 | 1 |  |  | 1 |  |  | 0.26 |  | 1 |  |  | 1 |  |  | 0.34 |
| ≦ 20% | 0.82 | (0.46,1.46) |  | 0.47 | (0.24,0.94) |  |  |  | 0.68 | (0.42,1.11) |  | 0.96 | (0.58,1.57) |  |  |
| > 20% | 2.87 | (1.71,4.82) |  | 1.58 | (0.92,2.72) |  |  |  | 1.06 | (0.62,1.82) |  | 1.60 | (0.97,2.66) |  |  |
| p value for trend | 0.001 |  |  | 0.30 |  |  |  |  | 0.77 |  |  | 0.11 |  |  |  |
